# Socioeconomic impact relating to clinical condition on Pandemic (H1N1) Influenza

**Published:** 2011-01-10

**Authors:** T Manabe, AL Higera-Iglesias, J Takasaki, S Izumi, ME Vazquez-Manriquez, K Kudo

**Affiliations:** 1 Disease Control and Prevention Center, National Center for Global Health and Medicine, Shinjuku-ku, Tokyo, 162-8655 Japan; 2 Research Center for Clinical Epidemiology, National Institute of Respiratory Diseases, Mexico D.F., Mexico; 3 Department of Pathology, National Institute of Respiratory Diseases, Mexico D.F., Mexico

## Background

In April 2009, Pandemic (H1N1) 2009 has reported from Mexico and has spread so rapidly in the world-wide. However, the number of dead cases caused by this infection has a big difference among the counties and regions. We describe the considerable reasons why first Nations people have experienced such high rate of severe conditions from the socioeconomic point of view which are related to healthcare.

## Methods

The four categorized population in Mexico by their healthcare insurance which related to their economic status and living environment were analyzed focusing on their health behavior in the first wave of Pandemic H1N1 2009 was occurred in Mexico. Also, we surveyed the socioeconomic factors for the dead cases who hospitalized in National Institute of Respiratory Diseases (INER), Mexico and their behaviors relating to Pandemic (H1N1) 2009. Based on those data, we evaluated factors which might influence to their clinical conditions. Those aspects were discussed with the comparison with Japan where the total number of dead case was small.

## Results

Pandemic (H1N1) 2009 was first announced from Mexico where had insufficient provision preparedness under this circumstance. However, poverty, the large uninsured population (about 60% of population), and the low sanitary level were evaluated as the factors which influenced to the clinical conditions as well as difficulty in information transmission in the country that has 65 indigenous minority languages as “national languages, along with Spanish.” The areas with the large number of dead cases by the first 3 months matched with the area of rural poverty in Mexico. On the other hand, Japan has matured universal national health insurance system and only 0.2% of uninsured population. In addition, faster access to healthcare is their tradition when people gets symptoms and they have high sanitary knowledge including using masks.

## Conclusion

Self economic condition, health insurance status, limited sanitation, and lack of information lead to the less access to the healthcare which resulted in delayed of medical intervention including anti-viral treatment. It is difficult to eradicate poverty in Mexico and the health insurance situation in the short period of time. However, proposing the advantage of Japan such as prompt information, and sanitary behavior will help to increase their chance to get early medical intervention.

